# Multiple myeloma presenting as an intramedullary spinal cord tumor: a case report and review of the literature

**DOI:** 10.1186/s13256-020-02496-5

**Published:** 2020-10-16

**Authors:** Long Di, Kevin Huang, Tigran Kesayan, Derek Kroll, Rachid C. Baz, Robert J. Macaulay, Nam D. Tran

**Affiliations:** 1 Department of Neurosurgery, University of South Florida, Tampa, FL USA; 2 Department of Neuro-Oncology, Moffitt Cancer Center, 12902 USF Magnolia Drive, Tampa, FL 33612 USA; 3 Department of Neurology, University of South Florida, Tampa, FL USA; 4 Department of Hematology and Oncology, Moffit Cancer Center, Tampa, FL USA; 5 Department of Pathology, Moffit Cancer Center, Tampa, FL USA

**Keywords:** Multiple myeloma, Intramedullary spinal cord neoplasm, Metastasis, Spinal cord, Case report

## Abstract

**Background:**

Extramedullary disease in multiple myeloma often portends a worse diagnosis. In approximately 1% of cases, multiple myeloma may metastasize to the central nervous system as either leptomeningeal involvement or an intracranial, intraparenchymal lesion. Spinal cord metastases, however, are exceedingly rare. We present a case of spinal cord multiple myeloma as well as a literature review of reported cases.

**Case presentation:**

A 66-year-old African American man with multiple myeloma presented with acute midthoracic pain and lower extremity paresis and paresthesia. Magnetic resonance imaging of the spine revealed two contrast-enhancing intramedullary enhancing lesions in the T1–T2 and T6–T7 cord. Resection with biopsy yielded a diagnosis of metastatic multiple myeloma.

**Conclusion:**

To date, only six cases of extramedullary disease to the spinal cord in patients with multiple myeloma have been reported, including our patient’s case. In all cases, neurologic deficit was observed at presentation, and magnetic resonance imaging of the spine revealed an intramedullary, homogeneously enhancing lesion. Current evidence suggests worse prognosis in patients with extramedullary disease to the central nervous system, and treatment paradigms remain debatable.

## Introduction

Multiple myeloma (MM) is a hematologic malignancy that accounts for approximately 1.6% of all cancer cases diagnosed in the United States [1]. Extramedullary hematopoietic (EMH) MM, occurring outside of the bone marrow, often portends a poor prognosis and may rarely involve the central nervous system (CNS), causing neurologic deficit, disability, and diminished quality of life [2, 3]. CNS EMH typically presents as an intracranial metastasis that is postulated to arise from hematogenous spread or contiguous seeding from local lytic bone lesions [4, 5]. However, intramedullary spinal cord metastases are exceptionally rare. We present a case of a patient with MM and EMH in the thoracic spinal cord and provide a comprehensive review and discussion of previously reported cases.

## Case presentation

A 66-year-old African American man with relapsed refractory MM and peripheral neuropathy presented to our neurosurgery clinic with a 1-day history of sharp, nonradiating midthoracic back pain and associated numbness and weakness in both legs. He had difficulty ambulating. He had been diagnosed with MM about 3.5 years ago, when he presented with bony pain and imaging revealed lytic bone lesions. His bone marrow biopsy at the time was notable for 70% involvement with MM. Gene expression profiling revealed a high-risk CD-1 subtype, and the result of fluorescence *in situ* hybridization was notable for t(11;14). He was diagnosed with kappa light chain, International Staging System stage 1, Durie-Salmon stage IIIA MM. Over the past 3.5 years, he had received five prior lines of therapy, including bortezomib, lenalidomide, pomalidomide, carfilzomib, daratumumab, high-dose melphalan, autologous stem cell transplant, and venetoclax. In one of his prior progressive disease events, he was noted to have extramedullary disease with subcutaneous plasmacytomas.

**Fig. 1:**
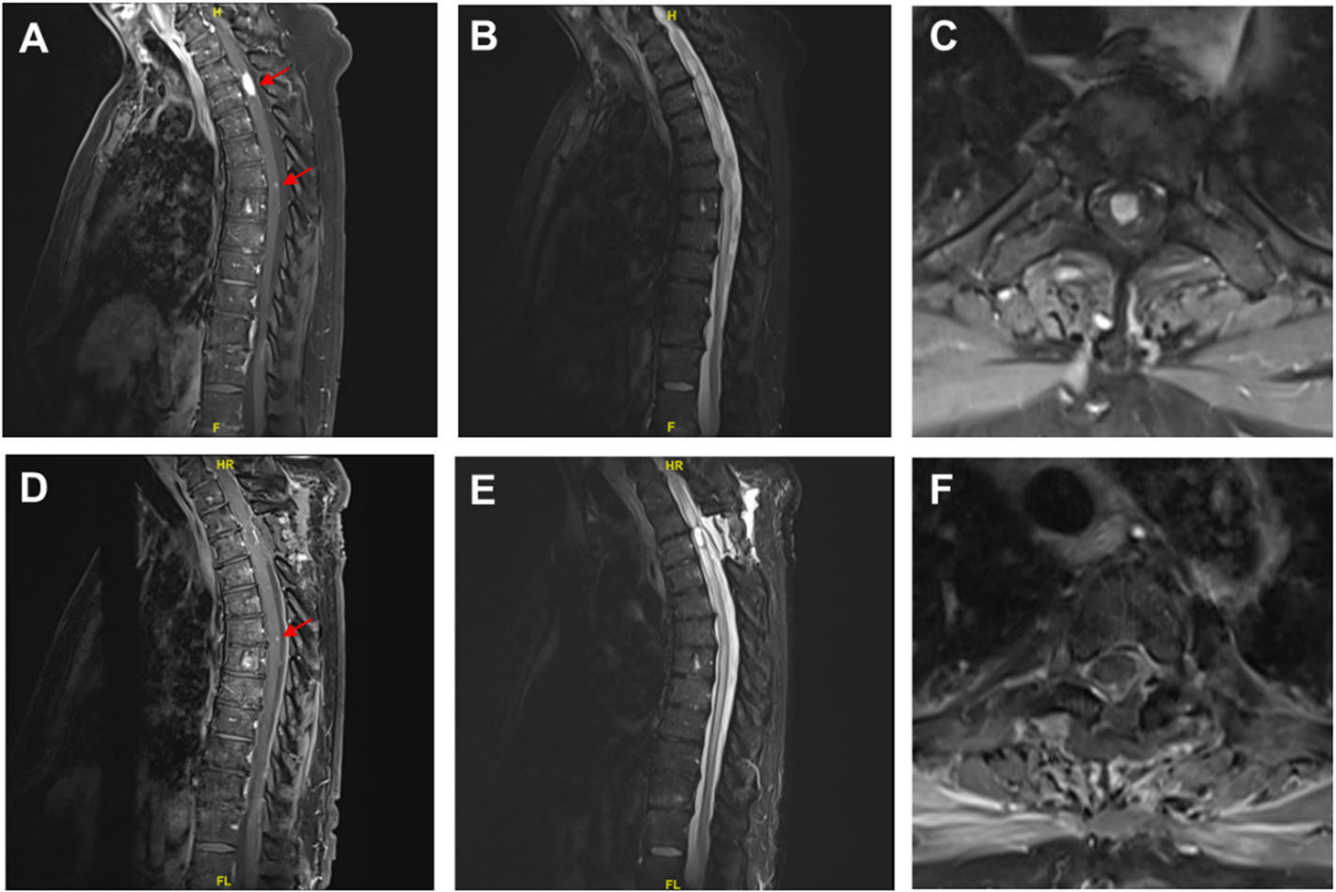
**a**–**c** Post-contrast sagittal T1-WI (T1-WI + contrast), T2-WI STIR, and axial T1-WI + contrast images showing intramedullary metastatic lesion (red arrows). Sagittal sequences show a dramatically enhancing lesion at the T2–T3 vertebral level and a second enhancing lesion in the dorsal cord at T6–T7. Axial sequences confirm the presence of the T2–T3 intramedullary spinal cord lesion. **d**–**f** Post-operative sequences showing complete resection of the T2–T3 lesion (red arrow). Expected post-surgical changes are seen with associated spinal cord edema. The T6–T7 lesion was not resected and remains identifiable on T1-WI + contrast magnetic resonance image. *STIR* short tau inversion recovery, *WI* weighted image

The patient’s motor examination revealed 4/5 strength in hip flexion and knee extension bilaterally. His patellar and Achilles deep tendons reflexes were 1+ bilaterally. His sensation to light touch was intact but subjectively decreased in a patchy distribution below the T5–T6 dermatome. On the basis of these findings, magnetic resonance imaging (MRI) of the thoracic spine was performed, which revealed two contrast-enhancing intramedullary lesions, with the largest at the T2–T3 level and a smaller lesion at T6–T7 (Fig. 1a–c). The result of MRI of the brain with and without contrast was unremarkable.

A working diagnosis of a neoplastic process was made. Given the rarity of MM with CNS metastasis, a biopsy for pathologic analysis was recommended. The patient underwent a thoracic T2–T3 laminectomy and intradural exploration. The spinal cord appeared mildly expanded. Ultrasound was used to localize the intramedullary tumor. Using standard microsurgical technique, a midline myelotomy was performed and immediately revealed a tan, well-circumscribed mass. The tumor was circumferentially mobilized, and complete tumor resection was performed (Fig. 1d–f).

The histological diagnosis yielded metastatic MM with high cellularity, amphophilic cytoplasm, rounded cell borders, and irregular pleomorphic nuclei. The myeloid component was demarcated from CNS tissue consistent with spinal cord. Numerous mitotic figures were observed, up to seven per high-power field. The neoplastic cells were immunoreactive for CD138 with only scattered overexpression of p53 interpreted as physiological upregulation. The patient’s Ki67 index was 75%. *In situ* hybridization revealed strong positive expression of kappa light chain with minimal lambda staining.

Postoperatively, the patient’s motor function improved from his preoperative baseline with mild worsening of proprioception. His neurologic examination result remained stable at 3 months. He completed fractionated stereotactic radiosurgery with 2 Gy in eight fractions.

## Discussion

We present a case of a patient with MM and EMH with metastasis to the thoracic spinal cord causing intramedullary disease. In addition, we conducted a literature review of all reported cases of MM or plasmacytoma with spinal cord metastasis. The results are summarized in Table 1. Including our patient’s case, there exist only six reported cases, four diagnosed as MM and two diagnosed as plasmacytoma. Five of the six patients were male (83.3%), and one patient was female (16.67%). Mean age at presentation was 51.2 years old. The most common presenting neurologic deficit was muscle weakness, which occurred in four of six cases (66.7%), followed by paresthesia in two of six cases (33.3%). In all cases, lesions were contrast-enhancing on either T1- or T2-weighted MRI sequences.

**Table 1:** Clinical results of multiple myeloma or plasmacytoma metastasizing to the spinal cord in the literature

Study	Year	Age (years)	Sex	Diagnosis	Neurologic deficits	Tumor location	MRI features	Treatment	Overall survival (months)
Di *et al.*	2019	66	M	Multiple myeloma	Lower extremity paresthesia and weakness, gait difficulty	T2–T3, T6–T7	T1 w/contrast: enhancing intramedullary mass with prominent associated spinal cord edema	Surgery + RT	N/A
Varettoni *et al*.	2008	56	M	Multiple myeloma	Weakness, paraparesis	Thoracic (T1–T2, T5–T6) and lumbar (L2–L3)	T1-weighted: progression of bone lesions, paraspinal plasmacytoma, and diffuse infiltration of the spinal cord	Chemo-RT	1.4
Hans *et al*.	2013	52	M	Plasmacytoma	Paresthesia, sensory deficit, progressive tetraparesis	C5–C6	T1/T2 showed mild enlargement of the cord with slight signal intensity from C5–C6. T2 w/contrast enhancement showed small, irregular area of “mild to moderate nodular homogeneous contrast enhancement” at ventral periphery of C5	Chemo-RT	Not reported, but describes significant neurologic deterioration at 10 months
Vale *et al*.	2012	51	M	Multiple myeloma	Weakness and paresis of left lower extremity	L1–cauda equina	Sagittal T2 w/contrast showed diffuse infiltration of the cauda equina, extending from L1 to L4. Axial T2 w/contrast showed enhancement of roots at L3 level.	Chemo-RT	11
Touzeau *et al*.	2004	51	F	Multiple myeloma	Progressive ataxia	Multiple lesions from C2 to T6	N/A	Chemo-RT	6.75
Gao *et al*.	2007	31	M	Plasmacytoma	Progressive lower extremity weakness and abasia. Bilateral abdominal, cremasteric, patellar tendon,and Achilles tendon reflexes absent	T7–T8	T1-weighted: extensive homogeneous isointense signal T6–T10T2-weighted: high-signal T6–T10T1 w/contrast: enhancing irregular lesion in anterior portion of T7–T8Chest, thoracic, and lumbar spine normal on MRI	Surgery	Not reported

*Abbreviations: MRI* magnetic resonance imaging, *N/A* not applicable, *RT* radiotherapy

### Differential diagnosis of MM

Diagnosing MM often requires the evaluation of clinical, radiographic, histopathologic, and laboratory findings [6–8]. In symptomatic patients, metastasis to the axial skeleton most often presents with back pain, vertebral fractures, or paresthesia and paresis due to spinal cord compression [9]. Lytic bone lesions may result in hypercalcemia, and renal dysfunction may present as anemia and proteinuria [10]. The differential diagnosis of MM may include monoclonal gammopathy of uncertain significance, Waldenstrom macroglobulinemia, and other plasma cell dyscrasias. The CRAB acronym (calcium elevation, renal dysfunction, anemia, and bone disease) was established by the International Myeloma Working Group to summarize the aforementioned clinical manifestations of MM as well as to differentiate between MM and similar plasma cell dyscrasias such as solitary plasmacytoma [11]. Differentiating MM from solitary plasmacytoma involves review of radiographic imaging and bone marrow biopsy [1]. Both dyscrasias require biopsy of bone lesions with evidence of clonal plasma cells. However, in solitary plasmacytoma, the CRAB symptoms are absent; there is no evidence of clonal plasma cells on bone marrow biopsy; and radiographic imaging reveals no other abnormalities aside from the primary lesion.

### EMH disease in MM

EMH is relatively uncommon at diagnosis but may occur later in the disease progression or at the time of relapse. Incidence of EMH in newly diagnosed MM is approximately 7–18% [12–14]. This proportion increases to 6–20% late in the disease course [13–16]. Several studies have shown that patients with EMH at presentation have significantly shorter survival with conventional chemotherapy [2, 12, 13]. In addition, Pour *et al.* described significantly worse outcomes in patients with soft tissue–related EMH than in bone-related EMH, with median survival rates of 5 months and 12 months, respectively (*P* = 0.022) [2]. Median survival of patients with CNS EMH shows similarly poor outcomes, with most studies reporting median survival of 2–8 months [3, 4, 17–22]. However, several of these studies included only intracranial metastases, and the others did not specify location further than detailing CNS involvement. Due to the extreme rarity of EMH involving the spinal cord, it is difficult to determine whether patient survival may differ from intracranial involvement. In consideration of the small sample size, we report an overall survival of 1–11 months on the basis of our literature review.

### MM in the CNS

Involvement of the CNS is relatively uncommon, with approximately 1–2% of patients with MM exhibiting a secondary CNS malignancy [23, 24]. Most often, these occur as either an intraparenchymal or meningeal lesion. MM metastasis to the spinal cord is exceedingly rare. Our review returned a total of six reported patients with metastatic, intramedullary spinal cord MM or plasmacytoma in the past 15 years, including our patient’s case. Five patients were male (83.3%), and one patient was female (16.7%). Two cases had a final diagnosis of plasmacytoma (33.3%), and four had a diagnosis of MM (66.7%). Bence-Jones proteinuria was reported in two cases (33.3%). The level of spinal cord metastasis varied from the cervical cord to the cauda equina.

Diagnosis of MM in the CNS often involves contrast-enhanced MRI of the head and/or spine. It should be noted that there remains concern for the use of iodine-based contrast agents in MM and monoclonal gammopathies [25]. Many radiologists consider the use of iodinated contrast material to be contraindicated in the setting of MM due to impaired renal function. Following administration of gadolinium contrast, MM may present as diffuse leptomeningeal enhancement or punctate, intraparenchymal lesions [26]. MM of the spinal cord shares similar characteristics. In all cases in which radiologic findings were reported, contrast-enhanced T1 weighted image (T1-WI) MRI of the spine showed a contrast-enhancing lesion; these lesions can present diffusely, with multiple enhancing lesions spread across spinal levels.

### Pathogenesis of CNS EMH

Several theories have been posited to suggest how CNS involvement in MM may arise. Local paraskeletal seeding of meninges and subsequent invasion into neural tissue has been suggested. Gozzetti *et al.* found neuroimaging evidence of contiguous spread arising directly from adjacent bone lesions [4]. Another hypothesis suggests hematogenous spread from traversal of the arachnoid veins by myeloma cells [5]. Spillage of cells into the cerebrospinal fluid would thus then be evident on cytologic assessment [20]. In this way, the neural invasion of MM closely reflects that seen in acute lymphoblastic leukemia, in which CNS involvement initially involves the adventitia bordering the arachnoid veins [27]. Few studies have investigated the molecular basis of CNS myeloma, but the acquisition of p53 gene mutations seems to be associated with advanced forms of disease [28, 29]. In a sample of nine patients, Chang *et al.* identified an 89% rate of p53 deletions, which stands in stark contrast to 10–15% of patients with MM who harbor p53 deletions but do not have CNS involvement [29]. In our patient’s case, immunostaining revealed only scattered p53 overexpression, suggestive of physiological upregulation rather than a gene mutation. Multiple lytic and lucent osseous lesions throughout the spinal axis were identified, notably in the thoracic spine. These were attributed to either multiple osseous degenerative changes or multifocal myelomatous disease. Thus, it is possible that the intramedullary disease in our patient arose from contiguous spread from nearby bone lesions. However, the presence of the second T6–T7 lesion located dorsally along the cord favors hematogenous spread from penetrating branches of the posterior spinal arteries, because contiguous spread throughout the width of the spinal cord from a vertebral lesion seems less likely.

### Treatment paradigms for CNS MM

Treatment of CNS MM is not well defined; however, some studies have suggested systemic therapy, occasionally with adjuvant radiation [20]. Systemic strategies present challenges because the blood–brain barrier (BBB) precludes treatment with traditional chemotherapeutics, such as high-dose melphalan, cyclophosphamide, proteasome inhibitors, or monoclonal antibodies. High-dose methotrexate or cytarabine is effective in penetrating the CNS but is ineffective against MM [30]. Thus, effective therapy necessitates good BBB permeability as well as action against MM. Thalidomide, lenalidomide, and pomalidomide have been shown to traverse the BBB in primates [31], and combination thalidomide and bendamustine has been shown to achieve a robust effect [32]. Of course, its use has yet to be validated in large-sample studies. More recently, selinexor was approved for patients with advanced MM, and this agent seems to cross the BBB. Combination BRAF and mitogen-activated protein kinase kinase 1/2 inhibitors dabrafenib/trametinib have also been employed to good response; however, recent evidence suggests potential mechanisms of drug resistance [33]. Given the reported radiosensitivity of plasma cell dyscrasias, concurrent radiotherapy may be considered [4, 34, 35]. Ultimately, intrathecal chemotherapy with a systemic anti-MM immunomodulatory regimen and cranial and/or spinal irradiation seems an ideal approach to management [21, 30]. Of six patients in our review, three patients (50%) were treated with chemoradiation therapy (chemo-RT); two patients (33.3%), including our own, underwent resection with adjuvant chemo-RT; and one patient (16.7%) underwent biopsy with chemo-RT.

All patients treated with chemo-RT saw progression in paresis and other neurological deficits over the course of treatment. Reported overall survival ranges from 1.4 to 11 months among patients treated with chemo-RT only. The role of surgery in intracranial EMH is unclear, and resection is not frequently performed [20]. However, in spinal cord metastasis, resection may assume a more prominent role because mass effect from intramedullary lesions may perturb motor and sensory tracts and compress nerve roots, contributing to radiculopathies. We found no data for patients treated with resection and chemo-RT. Hans *et al.* noted that surgical excision should be considered whenever possible. However, it is difficult to interpret the effect of surgery on patient morbidity and mortality, given the paucity of data. Our patient did not experience any perioperative complications, but the long-term effect of surgery on disease progression has yet to be observed.

## Conclusion

Intramedullary spinal cord metastasis is exceedingly rare and may present as paresthesia and myelopathy in patients with a history of MM. Intramedullary metastases appear as moderately contrast-enhancing lesions on T1-weighted images, often with diffuse infiltration across multiple spinal levels. Surgical debulking should be considered to alleviate mass effect on white matter tracts and nerve root compression. Radiotherapy with systemic therapy that ideally has BBB penetration remains a mainstay of treatment for managing this complicated stage of disease.

## Data Availability

The de-identified patient information presented in this report is available from the corresponding author on reasonable request.
